# Effects of Apricot Fibre on the Physicochemical Characteristics, the Sensory Properties and Bacterial Viability of Nonfat Probiotic Yoghurts

**DOI:** 10.3390/foods8010033

**Published:** 2019-01-18

**Authors:** Oya Berkay Karaca, Nuray Güzeler, Hasan Tangüler, Kurban Yaşar, Mutlu Buket Akın

**Affiliations:** 1 Karatas School of Tourism and Hotel Management, Cukurova University, 01903 Adana, Turkey; 2 Agricultural Faculty, Department of Food Engineering, Cukurova University, 01330 Adana, Turkey; nguzeler10@gmail.com; 3 Faculty of Engineering, Department of Food Engineering, Nigde University, 51245 Nigde, Turkey; htanguler@nigde.edu.tr; 4 Department of Food Engineering, Osmaniye Korkut Ata University, 80000 Osmaniye, Turkey; kurbanyasar@osmaniye.edu.tr; 5 Faculty of Engineering, Department of Food Engineering, Harran University, 63100 Şanlıurfa, Turkey; mutluakin@harran.edu.tr

**Keywords:** apricot fibre, *Bifidobacterium* BB-12, *L. acidophilus* LA-5, lactic and acetic acids, probiotic yoghurt

## Abstract

In this study, the physical, chemical, rheological, and microbiological characteristics and the sensory properties of nonfat probiotic yoghurt produced at two different concentrations of apricot fibre (1% and 2%, *w*/*v*) and three different types of probiotic culture (*Lactobacillus* (*L.*) *acidophilus* LA-5, *Bifidobacterium animalis* subsp. *lactis* BB-12 (*Bifidobacterium* BB-12), and their mixtures) were investigated. As the fibre content increased, the rheological, structural, and sensory properties of probiotic yoghurt were negatively affected, while counts of *L. delbrueckii* subsp. *bulgaricus*, *L. acidophilus* LA-5, and *Bifidobacterium* BB-12 increased. When all the results were evaluated, the best results were obtained by using *L. acidophilus* LA-5 as probiotic culture and adding 1% (*w*/*v*) apricot fibre.

## 1. Introduction

Consumers across the world are becoming more interested in foods with health-promoting features as they gain more awareness of the links between food and health. Among functional foods, products containing probiotics are showing promising trends worldwide [1]. Probiotics such as *Lactobacillus* and *Bifidobacterium* spp. are bacterial members of the human gut microbiota that exert several beneficial effects on human health and well-being through the production of short-chain fatty acids, which improves the intestinal microbial balance, resulting in the inhibiting bacterial pathogens, reducing colon cancer risk, stimulating the immune system, and lowering serum cholesterol levels [2]. In order to produce therapeutic benefits, a suggested minimum level for probiotic bacteria in fermented milk is above 10^6^ cfu mL^−1^ [3]. Several factors are responsible for the viability of these organisms, e.g., the strains used, growth conditions, antagonism among cultures present, storage time and temperature, initial counts, hydrogen peroxide and oxygen contents in the medium, and the amount of organic acids in the product [4]. Considerable studies have been conducted to stimulate the growth of probiotic bacteria during yoghurt fermentation and to improve their survival until the use-by date, by supplementing yoghurt milk with growth factors such as vitamin-enriched protein hydrolysate, amino nitrogen whey protein concentrate and cysteine [5]. A recent approach is to incorporate prebiotic substrates to support the growth and activity of probiotics [6,7].

Apricot is a rich source of sugars, fibres, minerals, bioactive phytochemicals, and vitamins like A, C, thiamine, riboflavin, niacin, and pantothenic acid. Among the phytochemicals, phenolics, carotenoids, and antioxidants are important for their biological value [8]. The aim of this study was to investigate the effects of apricot fibre (AF) on the overall quality and viable bacteria counts in nonfat probiotic yoghurt in order to set up the best formulation in the supplementation of food-grade fibre. For this purpose, nonfat probiotic yoghurts were manufactured by adding different rates of AF and individual and mixture cultures of *Lactobacillus acidophilus* LA-5 and *Bifidobacterium animalis* subsp. *lactis* BB-12.

## 2. Materials and Methods

### 2.1. Materials

Raw cow’s milk used in the experiments was obtained from the Animal Husbandry section of the Agricultural Faculty. Lyophilised starter cultures (coded FYS11, Marshall, France) containing *Streptococcus* (*Str.*) *thermophilus* and *Lactobacillus* (*L.*) *delbrueckii* ssp. *bulgaricus* were used as starter culture. In addition, lyophilised *L. acidophilus* LA-5 and *Bifidobacterium animalis* subsp. *lactis* BB-12 cultures were obtained from CHR-Hansen Company (Hørsholm, Denmark). All bacteria were maintained on de Man, Rogosa and Sharpe (MRS) agar slants. Apricots were used as dietary fibre source, and they were obtained from Apricot Research Institute in the first week of July, when they had attained enough maturity.

### 2.2. Methods

#### 2.2.1. Apricot Fibre Production

Fresh apricots were washed, cut lengthwise, and their kernel was removed. The apricot pieces were added to water containing citric acid (1%, *w*/*v*) to avoid any browning. They were taken from the water containing citric acid, and their water was removed. They were placed in freezer bags in the refrigerator. Then, they were dried in a freeze dryer (Ilshin, FD-8512, ilShin Biobase Europe B.V., Kryptonstraat 33, Netherlands) at −70 °C (condenser temperature) with 5 mTorr of pressure and a total time cycle of 48 h (freeze-drying time). Dried apricots were broken into powder in a blender (Heidolph Diax 900, Merck KGaA, Darmstadt, Germany) and sieved to remove large pieces. The obtained apricot fibre (moisture 5%, fat 0.40%, protein 4.00%, ash 3.80%, sugar 90.40%, cellulose 4.00%) was stored in closed plastic containers in a freezer at −20 °C.

#### 2.2.2. The Production of Yoghurt Contains Apricot Fibre Using Probiotic Culture

The fat content in the cow’s milk that was brought to the dairy technology laboratory was adjusted to 0.1% (*v*/*v*) using a cream separator (Elecrem, Vanves, France). Then, the milk was divided into seven parts, and milk powder and apricot fibre were added at the different levels given in Table 1. After blending the milk with milk powder and apricot fibre, the mixtures were separately homogenised using an Ultra Turrax blender (IKA, Merck, Germany) at 14,000 rpm until all ingredients were dissolved. Then, the homogenates were pasteurised at 85 °C for 5 min and cooled to 45 ± 1 °C. Starter culture (3%, *v*/*v*) and probiotic culture were added at a rate of 3% (about 10^6^ cfu mL^−1^; 1:1) to the cooled milks and filled into plastic yoghurt cups (200 mL). The samples were incubated in a Medcenter incubator (Friocell, Planegg/München, Germany) at 43 ± 1 °C until pH 4.7. At the end of incubation, all yoghurt samples were stored at refrigerator temperature (4 ± 1 °C) for 20 days. Yoghurt production was performed in triplicate. They were analysed after 1, 10, and 20 days of storage.

#### 2.2.3. Chemical Analysis

##### Analysis of Apricot Fibre

Total solids, protein, ash, and total amount of dietary fibre, fat contents, and sugar by soluble solids were determined according to Association of Official Analytical Chemists (AOAC) [9].

##### Chemical Analysis of Yoghurts

Total solids, fat, titratable acidity, protein, and ash [9] were measured. The pH values were measured using a digital pH meter (WTW, Wielheim, Germany). Lactic acid and acetic acid were analysed by an HPLC (Shimadzu LC-20AD, Shimadzu Corporation, Tokyo, Japan) using an Aminex HPX-87H column (Bio-Rad, Hercules, California, USA) at 50 °C. The eluent was 5 mmol L^−1^ H_2_SO_4_ in high-purity water at a flow rate of 0.6 mL min^−1^. Lactic acid and acetic acid amounts were calculated from a UV detector [10]. Standards (Merck, Darmstadt, Germany) were used to determine the concentration of organic acids. Replicates for all analytical determinations were carried out in duplicate.

##### Physical Measurements

Gel firmness was measured in the experimental yoghurts using a penetrometer model SUR BERLIN PNR 6 (Berlin, Germany) with a 15 g conical (45°) probe. Results were expressed as 1/10 millimetres of the penetration within 5 s. The viscosity values of the samples were determined using a Brookfield viscometer (model DV-II + Pro, Brookfield Engineering Laboratories, Middleboro, MA, USA) at 4 °C with a spindle (S64) rotation of 100 rpm and by applying a single constant shear rate (0.05 s^−^^1^). The readings were recorded at the 15th second of the measurement. The measurements were taken three times for each yoghurt sample, and the readings were recorded as centipoises. Water holding capacity (WHC) was determined using the centrifuge method with a modified procedure [11]. For this purpose, 5 g native yoghurt (NY) was centrifuged at 483× *g* for 30 min at 10 °C. After centrifugation, the supernatant was removed, and whey expelled (WE) was weighed and expressed as a percentage of yoghurt weight. Whey separation was considered as the amount of drained liquid (g) per twenty-five grams of sample. Each sample was weighed on a filter paper no. 589/2 (12.5 cm, 0.00009 g) placed on top of a funnel. The drainage time and temperature were 120 min and +4 °C, respectively [12]. The colour of the yoghurt samples was measured with a Minolta Chroma Meter CR-100 (Minolta, Osaka, Japan). L* (brightness, 100 = white, 0 = black), a* (+, red; −, green) and b* (+, yellow; −, blue) values were measured.

##### Microbiological Analysis of Yoghurts

For the counts of yoghurt and probiotic bacteria, 10 g of yoghurt samples were homogenised in 90 ml of peptone water (0.1% peptone) in a Stomacher 400 (Type BA7021, Seward Medical, Londan, UK) for at least 2 min. Samples were serially diluted in peptone water and spread inoculated (0.1 mL) onto plates. Plate counts of *Str. thermophilus* and *L. delbrueckii* subsp. *bulgaricus* were performed in M17 agar and MRS agar (Merck, Istanbul, Turkey), respectively. Incubations were conducted at 37 °C for 2 days (aerobically) and at 43 °C for 3 days (anaerobically), respectively. *L. acidophilus* LA-5 and *Bifidobacterium* BB-12 were enumerated on MRS-Sorbitol Agar (1% sorbitol) and MRS-NNLP Agar (100 mg neomycine sulphate, 15 mg nalidixic acid and 3 g LiCl), respectively [13,14]. The plates were incubated at 37 °C for 3 days (anaerobically). Anaerobic conditions were created using Anaerocult A sachets (Merck). Plates containing 20–200 colonies were counted, and the results were expressed as colony-forming units per gram (cfu g^−1^) of sample.

##### Sensory Characteristics

Sensory characteristics of the yoghurt samples were evaluated by a panel of ten expert members from the Laboratory of Milk and Dairy Products at Cukurova University according to a 0–5-point scale [15].

##### Statistical Analysis

Data were calculated for statistical significance by one-way analysis of variance (ANOVA). Means compared by Duncan’s test for statistical analysis were carried out. All analyses were made in triplicate and performed on the 1st, 10th, and 20th days of storage, except composition of milk and yoghurts.

## 3. Results and Discussion

### 3.1. Composition of Milk and Yoghurt

Titratable acidity (0.16 ± 0.01% LA), pH value (6.70 ± 0.00), and dry matter (8.81 ± 0.11%), fat (0.10 ± 0.00%), ash (0.74 ± 0.05%), and protein (3.33 ± 0.18%) contents of the nonfat milk used in the production of yoghurt were determined. The pH values, titratable acidity, and composition values of yoghurts were determined on the first day of storage and are given in Table 2. In the performed statistical evaluation, differences between titratable acidity values of yoghurts were found to be significant (*p* < 0.05). On the first day of storage, pH values, dry matter, protein, fat, and ash rates of yoghurts proved to be similar, whereas significant differences between titratable acidity values were found (*p* < 0.05). When the addition of apricot fibre rate increased so that dry matter did not change, milk powder and the total addition of ingredient rate was adjusted 6% (*w*/*v*). Garcia-Perez et al. [16], specified to no effect from the addition of orange fibre of yoghurt composition.

### 3.2. Changes in Chemical Properties of Probiotic Set Yoghurts during Storage

The pH, titratable acidity, and lactic and acetic acid changes of yoghurts during the storage period are given in Table 3. While pH values decreased considerably, titratable acidity values increased (*p* > 0.05). Additionally, yoghurts E, F, G, which had the highest added AF rate, had the highest values of titratable acidity after yoghurt A in general (*p* < 0.01). Lario et al. [17] specified that the addition of 1% (*w*/*v*) orange fibre to yoghurts caused a decrease in pH values of yoghurts and improved structural properties. 

In dairy products, lactic acid is one of major compounds of lactose degradation due to the lactic acid bacterial fermentation. During the fermentation of milk, depending on the microorganisms involved in the medium, lactic acid is produced via the glycolysis pathway while lactic and acetic acids are formed via the pentose phosphate pathway [18]. Lactic acid, which acts on milk protein, gives to yoghurt its texture and its characteristic sensory properties [19]. The highest amount of lactic acid was found in yoghurt A (without AF) on the first day of storage. The yoghurts produced with apricot fibre had lower levels of lactic and acetic acid than the control sample did on the first day of storage. In addition, lactic and acetic acid amounts decreased with increasing apricot fibre addition, and the effects of the addition of apricot fibre on the amount of lactic acid and acetic acid were significant (*p* < 0.01).

The amounts of lactic acid and acetic acid of yoghurts significantly increased while pH decreased significantly in yoghurts during the storage period (*p* < 0.05). Similar results were obtained by Ong et al. [20], who have stated that acetic acid amounts in cheddar produced by probiotic cultures increased during the storage period. *Str. thermophilus* and *L. delbrueckii* subsp. *bulgaricus* use lactose homofermentatively to produce lactic acid, whilst *Bifidobacterium* spp. produces lactic acid and acetic acid due to their heterofermentative nature by fermenting the same sugar [21]. As expected in the present study, lactic and acetic acid amounts in yoghurt samples produced with yoghurt starter cultures and *Bifidobacterium* BB-12 were higher than those in samples produced with yoghurt starter cultures and *L. acidophilus* LA-5 after 20 days of storage.

### 3.3. Changes in Rheological and Structural Properties of Probiotic Set Yoghurts

The changes in gel firmness, whey separation, water holding capacity, viscosity values during the storage period are given in Table 4. The effect of using apricot fibre on gel firmness values of yoghurts was found to be significant (*p* < 0.05). As the added AF rate increased, the titratable acidity values decreased, consequently decreasing gel firmness values, so yoghurts had a softer body. It was determined that gel firmness values of yoghurts with 2% (*w*/*v*) AF added were considerably different from those of yoghurts with 1% (*w*/*v*) AF on the 1st and 10th days of storage (*p* < 0.05). It is thought that the weakening of the gel structure is due to the addition of fibre instead of milk powder. Lario et al. [17] expressed that the rheological properties of yoghurt change related to added fibre ratios. The gel firmness values of yoghurts decreased during the storage period, and then it was observed that this decrease was significant in yoghurts A, E, and F (*p* < 0.05). The decrease of gel firmness values during the storage period arose from hydration of casein micelles of clot [22]. 

The least whey separation rate was in yoghurt C and the highest was in yoghurt G on the first day of storage. This alignment did not change on the 10th and 20th days of storage, that is, the highest whey separation rate was 2% in yoghurt G. In the case of the increasing rate of added AF and decreasing quantity of milk powder, it was determined that whey separation rates of yoghurts were increased (*p* < 0.01). A decrease in the amount of protein from the milk powder may lead to an increase in serum separation. Lario et al. [17] expressed that the changes of rheological properties of yoghurt are related to added fibre ratios. They reported that on the condition of 1% (*w*/*v*) orange fibre added to yoghurt, the quantity of whey separation was reduced and there were improved structural properties. In addition, Ferrandez Garcia and Gregor [23] informed that viscosity increased by rice and corn fibre addition to yoghurt, but it did not increase by sugar beet and soybean addition to yoghurt. The effect of different culture types on this feature was not statistically significant (*p* > 0.05). It was found that the whey separation values of all yoghurts decreased during the storage period, and this decrease was significant for yoghurts B, D, F, and G (*p* < 0.05). 

It was found that the water holding capacity of yoghurts had values close to each other on the first day of storage, and these rates decreased, for all yoghurts during storage (*p* > 0.05). It was determined that the water holding capacity of yoghurts was not affected by the addition of AF and by different probiotic cultures on the 1st and 10th days of storage (*p* > 0.05), and the water holding capacity of 2% (*w*/*v*) AF added yoghurts was affected considerably on the 20th day of storage (*p* < 0.05). Yoghurts F and G had the highest water holding capacity values. It can be stated that the result may be due to the high water-binding capacity of the fibres. Güler-Akın et al. [7] reported that the addition of apple fibre caused an increase in the water holding capacity of yoghurts.

Viscosity values were significantly affected by the added AF rate (*p* < 0.01). The viscosity values of yoghurts D and G produced with *L. acidophilus* LA-5 + *Bifidobacterium* BB-12 mixture culture were lower than those of other yoghurts produced with single culture. It was determined that the viscosity values of yoghurts increased considerably during the storage period (*p* < 0.01), and viscosity values of 1% (*w*/*v*) AF added yoghurts were higher than those of 2% (*w*/*v*) AF added yoghurts at this increase. The protein content which had an influence on the viscosity decreased with decreasing milk powder ratio in yoghurts; as a result, the viscosities of the yoghurts decreased. The highest value of viscosity was determined in the control yoghurt on the last day of storage (*p* < 0.01). Çayır [24] determined that the viscosity values of apricot puree added yoghurts increased during the storage period.

### 3.4. Changes in Colour Characteristics of Probiotic Set Yoghurts during Storage

L*, a*, b* values of yoghurts and their changes in time during the storage period are given in Table 5. The values of L* and a* were not influenced by culture types (*p* > 0.05). The L* values of yoghurts ranged between 87.09–90.32 on the first day of storage. The differences between L* values of yoghurts were determined to be very significant at all terms of storage time (*p* < 0.01). It was determined that the whiteness of yoghurt A (which has no AF added) was highest and L* values of yoghurts B, C, and D (which have 1% (*w*/*v*) AF added) were higher than yoghurts E, F, G (which have 2% (*w*/*v*) AF added), that is, when the AF increased, the values of L* decreased. The L* values of yoghurts at the end of storage decreased according to values on the first day, and this change was very significant in yoghurts B, E, and F (*p* < 0.05). Sanz et al. [25] specified that the brightness of yoghurts decreased by the addition of asparagus fibre, and yellow-green values increased. Garcia-Perez et al. [26] determined that orange fibre addition affected the colour of yoghurt: The L* value decreased, and the a* and b* values increased.

On the first day of storage, the a* values of yoghurts decreased (from −3.98 and −4.84) as the added AF rate increased. The difference between a* values of yoghurt A and the others is significant at all periods of storage (*p* < 0.01). It was found that the a* values of yoghurts F and G, which have the highest added AF rate, were lower than those of the control yoghurt on the 20th day of storage (*p* < 0.05). The a* values of yoghurts increased on the 20th day of storage compared to values on the first day of storage, so yoghurts had a greener colour by the end of storage. As the AF increased, the red colour increased in yoghurts as well. The storage period effect was not found to be significant on the a* values of yoghurt (*p* > 0.05).

As the added AF rate increased, b* values increased significantly. Two percent (*w*/*v*) AF added yoghurts had higher b* values on the first day of storage and during the storage period. The b* values of yoghurts increased during the storage period, and the added AF rate had a significant effect on the b* values of yoghurts during the storage period (*p* < 0.01). It was determined that changes in b* values were significant in the 2% (*w*/*v*) AF added yoghurts E, F, and G during the storage period (*p* < 0.01).

### 3.5. Bacterial Counts of Probiotic Set Yoghurts during Storage

Viable bacterial counts of yoghurt samples during the storage period are shown in Table 6. In spite of the sample F having the highest *Str. thermophilus* counts, added AF rate and using a different probiotic culture did not affect the number of *Str. thermophilus* (*p* > 0.05). The counts of *Str. thermophilus* decreased slowly during the storage period, and storage period had significant effect on the *Str. thermophilus* counts.

Usage of a different probiotic culture had no effect on the *L. delbrueckii* subsp. *bulgaricus* counts of yoghurt (*p* > 0.05). On the other hand, the addition of different rates of AF improved the viability of *L. delbrueckii* subsp. *bulgaricus* (*p* < 0.01). *L. delbrueckii* subsp. *bulgaricus* counts increased with increasing added AF rate. There was an approximately 1-log cycle decrease in the counts of *L. delbrueckii* subsp. *bulgaricus* at the end of storage (*p* < 0.01).

The culture type had significantly affected *L. acidophilus* LA-5 counts of samples (*p* < 0.01). The *L. acidophilus* LA-5 counts of yoghurt which was inoculated with a mixture of *L. acidophilus* LA-5 and *Bifidobacterium* BB-12 were higher than those of the yoghurt inoculated with individual *L. acidophilus* LA-5. Studies showed *Bifidobacterium* spp. and *L. acidophilus* LA-5 strains live in excellent symbiosis, and their counts were increased when they were inoculated together [27,28]. The addition of different rates of AF also improved the survival of *L. acidophilus* LA-5 (*p* < 0.01). The counts of *L. acidophilus* LA-5 increased as added AF rate increased due to the possible prebiotic effects of AF. Similar results were reported for many fibres by the other authors [29,30]. The counts of *L. acidophilus* LA-5 decreased during the storage period. The most important factors affecting the viability of *L. acidophilus* LA-5 are acidity and hydrogen peroxide [13]. The acidity of the samples increased during the storage period. During the storage period, although the viable counts of *L. acidophilus* LA-5 dropped in all samples, the counts in all probiotic yoghurts were found to be above the threshold for therapeutic minimum (10^6^–10^7^ cfu g^−1^).

The use of probiotic culture as individual or mixture had no effect statistically on the *Bifidobacterium* BB-12 (*p* > 0.05). However, Gomes and Malcata [31] reported that growth of *L. acidophilus* LA-5 caused to diminish the redox potential in the samples and so improved the counts of *Bifidobacterium* BB-12. On the other hand, the effect of different rates of AF was significant on the viability of *Bifidobacterium* BB-12 (*p* < 0.01). The counts of *Bifidobacterium* BB-12 increased as added AF rate increased (*p* < 0.01), but this increase was not enough to reach above 10^6^–10^7^ cfu g^−1^. *Bifidobacterium* BB-12 counts decreased during the storage period (*p* < 0.01), which could be attributed to antagonistic relationships between yoghurt bacteria and probiotic strains. At the end of storage, the counts of *Bifidobacterium* BB-12 decreased to approximately 1-log cycle in all probiotic yoghurts, and the counts were found to be below the threshold for therapeutic minimum (10^6^–10^7^ cfu g^−1^). Authors reported that some fibres, such as xylo-oligosaccharides, lactulose, rafinose, inulin, and citrus fibre had a prebiotic effect on the *Bifidobacterium* spp. [29].

### 3.6. Changes in Sensory Properties of Probiotic Set Yoghurt during Storage

**Figure 1:**
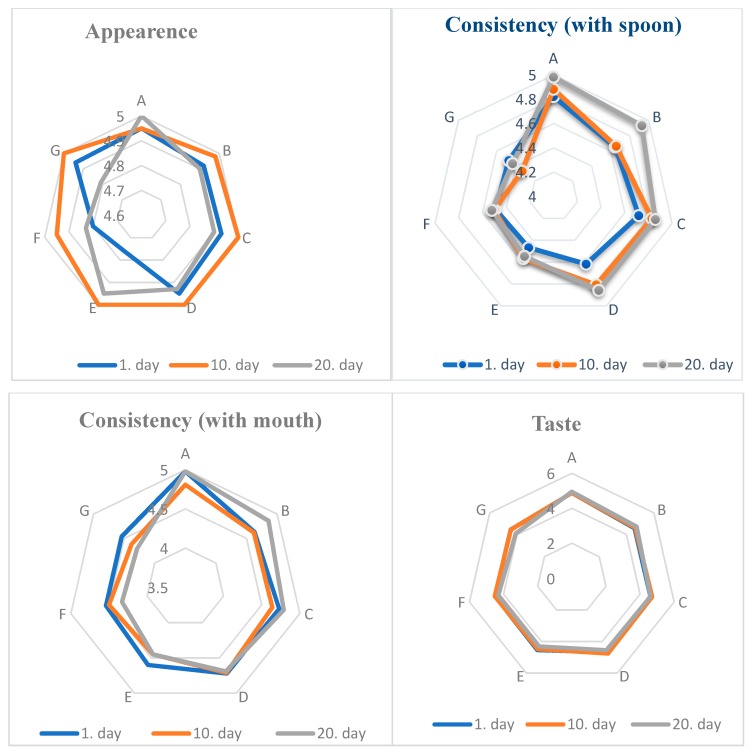
Sensory properties of probiotic set yoghurt during storage.

The scores recorded for appearance, consistency (by spoon and mouth), taste, and total sensory properties of yoghurts are given in Figure 1. The highest appearance score on the first day of storage was determined in the control yoghurt, and the lowest appearance scores were received by yoghurts F and G on the last day of storage (*p* > 0.05). Different fibre ratios did not affect appearance scores at all periods of storage (*p* > 0.05). While the consistency (with spoon) scores of yoghurts A and C were found to be the highest, yoghurts E and G had the lowest scores. It was determined that as the addition of AF increased and milk powder decreased, the consistency of scores of yoghurts with spoon decreased at all periods of storage (*p* < 0.01). This result can be explained by the protein content and the effect of the protein on the gel structure and is parallel to the results of gel firmness. On the first day of storage, while there was no difference between yoghurts, on the 10th and 20th days in the control yoghurt and the 1% (*w*/*v*) AF added yoghurt, consistency (with spoon) values were similar (*p* > 0.05) except for 2% (*w*/*v*) AF added yoghurts (*p* < 0.01).

As the added AF rate increased, the consistency score in mouth decreased. It was established that consistency scores with the mouth decreased according to first day values in yoghurts during the storage period. The effect of added AF rate was found to be significant on the difference between consistency scores of yoghurts (*p* < 0.05). The effect of storage time on the consistency scores with mouth was not significant except for yoghurt B (*p* > 0.05). The effect of added AF rate was not significant on the odour properties of yoghurts (*p* > 0.05), and as added AF rate increased, odour scores decreased.

The highest score of taste was yoghurt A without AF, and the lowest score was yoghurt G. While the difference between the flavour properties of yoghurt on the first day of storage was not found to be important, the control yoghurt and the 2% (*w*/*v*) AF added yoghurts E, F, and G were found to be significant and considerably different on the 10th and 20th days (*p* < 0.01). Generally, flavour scores of 1% (*w*/*v*) AF added yoghurts were the highest scores after those of yoghurt A. Additionally, 2% (*w*/*v*) AF added decreased the scores. During all periods of storage, yoghurt A had the highest total sensory score, and following this, the 1% (*w*/*v*) AF added yoghurts on the last day of storage did not change this order. It was determined that the effect of storage time was not significant on the odour, flavour, and total sensory scores of yoghurts (*p* > 0.05). The difference between the total sensory scores of yoghurts were very significant on the 10th and 20th days of storage (*p* < 0.05). Yoghurts containing individual cultures were found to have higher scores than those of their mixtures. Fernandez-Garcia and McGregor [23] specified that bamboo, wheat, and inulin fibre added yoghurts had sensory properties close to the control yoghurt. They informed that the sensory and rheological properties of apple fibre added yoghurt were different from those of control yoghurt. Additionally, Staffolo et al. [32] specified that bamboo, wheat, and inulin fibre added yoghurts had sensory properties close to control yoghurt despite apple fibre addition being different significant. 

## 4. Conclusions

The results of the analysis indicate that as the added AF rate increases, there is an increase in whey separation, gel firmness, water holding capacity, a* and b* values, *L. delbrueckii* subsp. *bulgaricus, L. acidophilus* LA-5, *Bifidobacterium* BB-12 counts of yoghurts, and a decrease in lactic and acetic acid amounts, the titratable acidity, viscosity, L* values, consistency, odour and flavour scores. The viscosity values of yoghurts D and G produced with *L. acidophilus* LA-5 + *Bifidobacterium* BB-12 mixture culture were lower than those of other yoghurts produced with single culture. The quantities of lactic and acetic acid using *Bifidobacterium* BB-12 probiotic culture were found to be higher on the first day and during the storage period. In the sensory evaluation, yoghurt A had the highest total sensory score, and following this, during all periods of storage, the 1% (*w*/*v*) AF added yoghurts. The usage of *L. acidophilus* LA-5 culture in yoghurts acquired more appreciation according to *Bifidobacterium* BB-12 and mixed cultures addition of yoghurt. According to the results, yoghurts containing individual cultures had higher scores than those of their mixtures and using *L. acidophilus* LA-5 as the probiotic culture and 5% milk powder +1% (*w*/*v*) AF addition for nonfat probiotic yoghurt production can be suggested.

## Figures and Tables

**Table 1:** Microorganisms and additives used in the production of yoghurt at different types.

Yog	AF (%)	MP (%)	Bacteria
**A**	0	6	yoghurt bacteria
**B**	1	5	yoghurt bacteria + *Lactobacillus acidophilus* LA-5
**C**	1	5	yoghurt bacteria + *Bifidobacterium* BB-12
**D**	1	5	yoghurt bacteria + *Lactobacillus acidophilus* LA-5 *+ Bifidobacterium* BB-12
**E**	2	4	yoghurt bacteria + *Lactobacillus acidophilus* LA-5
**F**	2	4	yoghurt bacteria + *Bifidobacterium* BB-12
**G**	2	4	yoghurt bacteria + *Lactobacillus acidophilus* LA-5*+Bifidobacterium* BB-12

Yog: Yoghurt; AF: Apricot fibre; MP: Milk powder; Yoghurt bacteria: *Streptococcus thermophilus* and *Lactobacillus* *delbrueckii* ssp. *bulgaricus*.

**Table 2:** Physicochemical composition of AF added nonfat yoghurts (*n* = 3).

	pH	Titratable Acidity (LA %)	Dry Matter (%)	Protein (%)	Fat (%)	Ash (%)
**Milk**	6.70 ± 0.00	0.160 ± 0.01	8.81 ± 0.11	3.33 ± 0.18	0.10 ± 0.00	0.74 ± 0.05
**A**	4.70 ± 0.07 ^a^	1.138 ± 0.14 ^a^	13.83 ± 0.40 ^a^	5.64 ± 0.71 ^a^	0.10 ± 0.00 ^a^	1.28 ± 0.09 ^a^
**B**	4.75 ± 0.06 ^a^	1.056 ± 0.09 ^a^^,^^b,c^	13.79 ± 0.18 ^a^	5.16 ± 0.92 ^a^	0.10 ± 0.00 ^a^	1.21 ± 0.04 ^a^
**C**	4.81 ± 0.05 ^a^	1.002 ± 0.05 ^b,c^	13.71 ± 0.22 ^a^	5.04 ± 0.27 ^a^	0.10 ± 0.00 ^a^	1.22 ± 0.07 ^a^
**D**	4.81 ± 0.08 ^a^	1.020 ± 0.05 ^b,c^	13.76 ± 0.12 ^a^	4.18 ± 0.54 ^a^	0.10 ± 0.00 ^a^	1.25 ± 0.08 ^a^
**E**	4.83 ± 0.13 ^a^	1.023 ± 0.07 ^b,c^	13.75 ± 0.13 ^a^	4.59 ± 0.34 ^a^	0.10 ± 0.00 ^a^	1.33 ± 0.25 ^a^
**F**	4.87 ± 0.17 ^a^	0.971 ± 0.07 ^c^	13.69 ± 0.18 ^a^	4.87 ± 0.23 ^a^	0.10 ± 0.00 ^a^	1.15 ± 0.03 ^a^
**G**	4.80 ±0.15 ^a^	1.090 ±0.08 ^a,b^	13.60 ± 0.23 ^a^	4.37 ± 0.59 ^a^	0.10 ± 0.00 ^a^	1.14 ± 0.02 ^a^

^a,b,c^ Means in the same column followed by different letters were significantly different (*p* < 0.05).

**Table 3:** Physicochemical properties of nonfat probiotic set yoghurts during storage (*n* = 3).

Day	Yoghurts A	Yoghurts B	Yoghurts C	Yoghurts D	Yoghurts E	Yoghurts F	Yoghurts G
**pH**							
1	4.70 ± 0.08 ^A,a^	4.75 ±.06 ^A,a^	4.81 ± 0.05 ^A,a^	4.81 ± 0.09 ^A,a^	4.83 ± 0.15 ^A,a^	4.87 ± 0.20 ^A,a^	4.80 ± 0.17 ^A,a^
10	4.54 ± 0.12 ^AB,a^	4.51 ± 0.21 ^A,a^	4.63 ± 0.17 ^AB,a^	4.60 ± 0.15 ^AB,a^	4.58 ± 0.09 ^B,a^	4.61 ± 0.04 ^AB,a^	4.52 ± 0.10 ^B,a^
20	4.37 ± 0.11 ^B,a^	4.41 ± 0.15 ^B,a^	4.45 ± 0.14 ^B,a^	4.47 ± 0.15 ^B,a^	4.42 ± 0.07 ^B,a^	4.41 ± 0.13 ^B,a^	4.37 ± 0.06 ^B,a^
Titratable acidity (LA%)							
1	1.138 ± 0.16 ^A,a^	1.056 ± 0.11 ^A,a^	1.002 ± 0.06 ^B,a^	1.020 ± 0.06 ^B,a^	1.023 ± 0.08 ^B,a^	0.971 ± 0.08 ^C,a^	1.090 ± 0.09 ^A,a^
10	1.192 ± 0.02 ^A,a^	1.182 ± 0.04 ^A,a^	1.150 ± 0.06 ^A,a^	1.165 ± 0.07 ^A,a^	1.197 ± 0.09 ^A,a^	1.102 ± 0.05 ^B,a^	1.125 ± 0.04 ^A,a^
20	1.272 ± 0.07 ^A,a^	1.153 ± 0.03 ^A,a^	1.243 ± 0.06 ^A,a^	1.233 ± 0.07 ^A,a^	1.265 ± 0.010 ^A,a^	1.243 ± 0.04 ^A,a^	1.250 ± 0.10 ^A,a^
Lactic acid (g/L)							
1	16.59 ± 0.04 ^C,a^	12.27 ± 0.08 ^C,b^	11.48 ± 0.23 ^C,d^	12.66 ± 0.21 ^C,b^	11.70 ± 0.15 ^B,cd^	12.94 ± 0.17 ^C,b^	12.03 ± 0.35 ^C,c^
10	18.70 ± 0.11 ^B,a^	14.38 ± 0.10 ^B,c^	14.42 ± 0.21 ^B,c^	14.41 ± 0.18 ^B,c^	12.31 ± 0.47 ^B,d^	14.42 ± 0.20 ^B,c^	14.51 ± 0.27 ^B,c^
20	19.48 ±0.06 ^A,a^	15.15 ± 1.40 ^A,bc^	16.42 ± 0.27 ^A,b^	15.53 ± 0.51 ^A,bc^	14.32 ± 1.03 ^A,c^	16.43 ± 0.36 ^A,b^	16.45 ± 0.08 ^A,b^
Acetic acid (g/L)							
1	0.792 ± 0.00 ^B,a^	0.595 ± 0.0 8 ^A,c^	0.518 ± 0.01 ^C,d^	0.697 ± 0.01 ^C,b^	0.439 ± 0.02 ^C,e^	0.708 ± 0.00 ^C,b^	0.574 ± 0.00 ^C,cd^
10	0.851 ± 0.02 ^A,a^	0.671 ± 0.05 ^A,b^	0.813 ± 0.03 ^B,a^	0.776 ± 0.03 ^B,a^	0.550 ± 0.03 ^B,c^	0.840 ± 0.02 ^B,a^	0.821 ± 0.07 ^B,a^
20	0.887 ± 0.04 ^A,b^	0.755 ± 0.05 ^A,c^	1.282 ± 0.11 ^A,a^	1.218 ± 0.03 ^A,a^	0.615 ± 0.00 ^A,d^	0.957 ± 0.04 ^A,b^	0.954 ± 0.00 ^A,b^

^a,b,c,d,e^ Means in the same row followed by different letters were significantly different (*p* < 0.05). ^A,B,C^ Means in the same column followed by different letters significantly different (*p* < 0.05).

**Table 4:** Rheological and structural properties of nonfat probiotic set yoghurts (*n* = 3).

Day	Yoghurts A	Yoghurts B	Yoghurts C	Yoghurts D	Yoghurts E	Yoghurts F	Yoghurts G
**Gel firmness (mm/5 sn)**							
**1**	196 ± 3.79 ^A,e^	196 ± 1.73 ^A,de^	203 ± 4.73 ^A,cd^	200 ± 3.79 ^A,cde^	206 ± 5.03 ^A,bc^	214 ± 2.52 ^A,a^	211 ± 2.89 ^A,ab^
**10**	195 ± 5.00 ^A,bcd^	194 ± 6.08 ^A,cd^	192 ± 5.03 ^A,d^	192 ± 3.79 ^A,cd^	201 ± 2.52 ^AB,abc^	203 ± 5.51 ^B,ab^	206 ± 3.22 ^A,a^
**20**	183 ± 4.51 ^B,d^	188 ± 4.04 ^A,cd^	191 ± 5.51 ^A,bc^	192 ± 4.16 ^A,bc^	196 ± 1.73 ^B,b^	195 ± 3.79 ^B,bc^	204 ± 4.16 ^A,a^
**Whey separation (%)**							
**1**	18.03 ± 2.08 ^A bc^	17.51 ± 1.96 ^A,bc^	15.43 ± 2.83 ^A,c^	20.04 ± 1.82 ^A,b^	24.11 ± 3.32 ^A,a^	23.95 ± 0.24 ^A,a^	24.64 ± 1.79 ^A,a^
**10**	15.19 ± 0.88 ^A^^,^^c^	16.89 ± 1.44 ^A,c^	14.73 ± 2.76 ^A,c^	17.28 ± 0.20 ^B,bc^	20.88 ± 2.69 ^A,a^	20.21 ± 1.42 ^B,ab^	22.56 ± 1.49 ^AB,a^
**20**	14.43 ± 1.85 ^A,c^	13.41 ± 0.20 ^B,c^	14.18 ± 0.30 ^A,c^	16.33 ± 0.20 ^B,b^	17.86 ± 1.26 ^A,b^	19.79 ± 0.21 ^B,a^	21.00 ± 0.50 ^B,a^
**Water holding capacity (%)**							
**1**	69.97 ± 2.35 ^A,a^	70.00 ± 2.21 ^A,a^	70.70 ± 1.99 ^A,a^	71.87 ± 1.63 ^A,a^	70.83 ± 4.46 ^A,a^	72.13 ± 3.51 ^A,a^	72.83 ± 2.73 ^A,a^
**10**	65.33 ± 3.41 ^A,a^	67.50 ± 2.44 ^A,a^	67.97 ± 2.57 ^A,a^	70.07 ± 2.84 ^A,a^	69.27 ± 1.72 ^A,a^	70.33 ± 1.83 ^A,a^	71.77 ± 2.87 ^A,a^
**20**	63.20 ± 3.73 ^A,b^	66.70 ± 1.21 ^A,ab^	67.43 ± 2.54 ^A,ab^	68.40 ± 2.71 ^A,a^	69.30 ± 2.43 ^A,a^	69.90 ± 1.74 ^A,a^	69.90 ± 1.35 ^A,a^
**Viscosity (100 cp)**							
**1**	2001 ± 77 ^C,b^	2150 ± 39 ^C,a^	1917 ± 37 ^C,bc^	1948 ± 82 ^C,bc^	2035 ± 21 ^B,b^	2009 ± 71 ^C,b^	1876 ± 84 ^C,c^
**10**	2876 ± 40 ^B,a^	2437 ± 48 ^B,b^	2422 ± 10 ^B,b^	2409 ± 71 ^B,b^	2355 ± 33 ^A,b^	2213 ± 58 ^B,c^	2151 ± 58 ^B,c^
**20**	3048 ± 11 ^A,a^	2906 ± 35 ^A,b^	2837 ± 21 ^A,c^	2595 ± 12 ^A,d^	2408 ± 66 ^A,e^	2391 ± 38 ^A,e^	2351 ± 50 ^A,e^

^a,b,c,d,e^ Means in the same row followed by different letters were significantly different (*p* < 0.05); ^A,B,C^ Means in the same column followed by different letters significantly different (*p* < 0.05).

**Table 5:** Colour properties in nonfat probiotic set yoghurts during storage (*n* = 3).

Day	Yoghurts A	Yoghurts B	Yoghurts C	Yoghurts D	Yoghurts E	Yoghurts F	Yoghurts G
L^*^							
**1**	90.32 ± 0.72 ^A,a^	89.22 ± 0.55 ^A,b^	89.46 ± 0.40 ^A,ab^	89.46 ± 0.56 ^A,ab^	87.74 ± 0.14 ^A,c^	87.76 ± 0.47 ^A,c^	87.09 ± 0.68 ^A,c^
**10**	90.12 ± 0.74 ^B,a^	87.37 ± 0.48 ^B,cd^	88.80 ± 0.61 ^A,b^	88.01 ± 0.83 ^A,bc^	86.20 ± 0.95 ^B,de^	85.34 ± 0.47 ^B,e^	86.44 ± 0.10 ^A,de^
**20**	90.29 ± 0.47 ^A,a^	88.33 ± 0.44 ^AB,bc^	87.76 ± 0.87 ^A,bcd^	88.62 ± 0.90 ^A,b^	86.83 ± 0.14 ^AB,d^	87.26 ± 0.90 ^A,cd^	86.52 ± 0.85A^d^
a^*^							
**1**	−4.84 ± 0.34 ^A,a^	−4.26 ± 0.31^A,b^	−4.34 ± 0.23 ^A,b^	−4.30 ± 0.15 ^A,b^	−4.01 ± 0.20 ^A,b^	−3.98 ± 0.25 ^A,b^	−4.00 ± 0.23 ^A,b^
**10**	−4.83 ± 0.27 ^A,a^	−4.07 ± 0.01^A,b^	−4.01 ± 0.02 ^A,b^	−4.06 ± 0.24 ^A,b^	−3.77 ± 0.14 ^A,bc^	−3.70 ± 0.22 ^A,c^	−3.80 ± 0.22 ^A,bc^
**20**	−4.70 ± 0.12 ^A,a^	−4.27 ± 0.14^A,b^	−4.14 ± 0.11 ^A,bc^	−3.95 ± 0.16 ^A,cd^	−3.90 ± 0.14 ^A,cd^	−3.87 ± 0.17 ^A,d^	−3.88 ± 0.10 ^A,d^
b^*^							
**1**	8.29 ± 0.89 ^A,d^	11.25 ± 0.83 ^A,bc^	11.06 ± 0.41 ^A,c^	11.08 ± 0.08 ^A,c^	12.34 ± 0.86 ^B,ab^	12.52 ± 0.25 ^B,a^	12.63 ± 0.73 ^B,a^
**10**	8.37 ± 0.65 ^A,c^	11.44 ± 0.15 ^A,b^	11.39 ± 0.34 ^A,b^	11.11 ± 0.79 ^A,b^	12.91 ± 0.47 ^B,a^	12.80 ± 0.81 ^B,a^	12.54 ± 0.65 ^B,a^
**20**	8.60 ± 1.06 ^A,d^	12.83 ± 0.78 ^A,b^	12.06 ± 0.94 ^A,bc^	11.48 ± 0.12 ^A,c^	14.18 ± 0.04 ^A,a^	14.18 ± 0.02 ^A,a^	14.74 ± 0.84 ^A,a^

^a,b,c,d,e^ Means in the same row followed by different letters were significantly different (*p* < 0.05); ^A,B,C^ Means in the same column followed by different letters significantly different (*p* < 0.05).

**Table 6:** The changes of viable bacteria counts of nonfat probiotic yoghurts during storage period (log cfu g^−1^) (*n* = 3).

Yoghurts	Storage Period (Day)	*S. thermophilus*	*L. delbrueckii* ssp. *bulgaricus*	*L. acidophilus* LA-5	*Bifidobacterium* BB-12
A	1	8.34 ± 0.08 ^a^	8.07 ± 0.01 ^abcde^	-	-
	10	7.87 ± 0.04 ^bc^	7.91 ± 0.06 ^de^	-	-
	20	7.73 ± 0.01 ^c^	7.28 ± 0.13 ^fg^	-	-
B	1	8.36 ± 0.11 ^a^	8.12 ± 0.04 ^abcd^	7.91 ± 0.05 ^a^	-
	10	7.76 ± 0.06 ^bc^	7.83 ± 0.03 ^e^	7.35 ± 0.03 ^d^	-
	20	7.72 ± 0.01 ^c^	7.26 ± 0.19 ^fg^	6.34 ± 0.03 ^g^	-
C	1	8.33 ± 0.07 ^a^	8.15 ± 0.10 ^abcd^	-	7.40 ± 0.07 ^a^
	10	7.85 ± 0.05 ^bc^	7.91 ± 0.05 ^de^	-	6.31 ± 0.11 ^b^
	20	7.77 ± 0.02 ^c^	7.33 ± 0.16 ^fg^	-	5.26 ± 0.02 ^d^
D	1	8.33 ± 0.06 ^a^	8.19 ± 0.04 ^ab^	7.96 ± 0.03 ^a^	7.47 ± 0.07 ^a^
	10	7.86 ± 0.06 ^bc^	7.43 ± 0.05 ^f^	7.50 ± 0.06 ^bc^	6.21 ± 0.10 ^b^
	20	7.72 ± 0.04 ^c^	7.13 ± 0.12 ^g^	6.56 ± 0.04 ^f^	5.43 ± 0.09 ^cd^
E	1	8.35 ± 0.14 ^a^	8.22 ± 0.08 ^a^	7.96 ± 0.01 ^a^	-
	10	7.92 ± 0.01 ^b^	7.91 ± 0.04 ^de^	7.41 ± 0.05 ^cd^	-
	20	7.73 ± 0.07 ^c^	7.42 ± 0.14 ^f^	6.65 ± 0.04 ^ef^	-
F	1	8.40 ± 0.10 ^a^	8.09 ± 0.04 ^abcd^	-	7.49 ± 0.06 ^a^
	10	7.90 ± 0.01 ^bc^	7.93 ± 0.03 ^cde^	-	6.40 ± 0.25 ^b^
	20	7.81 ± 0.05 ^c^	7.32 ± 0.17 ^fg^	-	5.64 ± 0.05 ^c^
G	1	8.33 ± 0.12 ^a^	8.17 ± 0.10 ^abc^	7.98 ± 0.02 ^a^	7.49 ± 0.05 ^a^
	10	7.90 ± 0.03 ^bc^	7.94 ± 0.01 ^bcde^	7.60 ± 0.04 ^b^	6.45 ± 0.09 ^b^
	20	7.72 ± 0.06 ^c^	7.28 ± 0.10 ^fg^	6.75 ± 0.03 ^c^	5.68 ± 0.06 ^c^

^a,b,c,d,e,f,g^ Different letters indicate significant differences among the samples (*p* < 0.01).
